# Detection of the *agr* System and Resistance to Antimicrobials in Biofilm-Producing *S. epidermidis*

**DOI:** 10.3390/molecules25235715

**Published:** 2020-12-03

**Authors:** Valéria Cataneli Pereira, Luiza Pinheiro-Hubinger, Adilson de Oliveira, Danilo Flávio Moraes Riboli, Katheryne Benini Martins, Letícia Calixto Romero, Maria de Lourdes Ribeiro de Souza da Cunha

**Affiliations:** 1 Sector of Microbiology and Immunology, Department of Chemical and Biological Sciences, Institute of Biosciences, UNESP—University Estadual Paulista, Botucatu CEP 18618-689, São Paulo, Brazil; valeriacataneli@gmail.com (V.C.P.); luizapinheiro@ibb.unesp.br (L.P.-H.); adilsonoliveiralp@ig.com.br (A.d.O.); daniloflaviomr@yahoo.com.br (D.F.M.R.); katheryne_bm@yahoo.com.br (K.B.M.); le.calixto22@gmail.com (L.C.R.); 2 Sector of Microbiology and Immunology, UNOESTE—University of West Paulista, Presidente Prudente CEP 19050-920, São Paulo, Brazil

**Keywords:** oxacillin resistance, ST2, *agr* locus, biofilm, antimicrobial resistance

## Abstract

The ability of *Staphylococcus epidermidis* to produce virulence factors, such as biofilm, added to its increased resistance to antimicrobials can cause infections that are difficult to treat. Many staphylococcal virulence factors are under the control of the accessory gene regulator (*agr*). The objective of this study was to establish the *agr* locus and susceptibility of biofilm-producing *S. epidermidis* specimens to antimicrobial agents, through PCR reactions, reverse transcription polymerase chain reaction (RT-PCR), and the determination of minimum inhibitory concentration (MIC), and to analyze the clonal profile of 300 strains isolated from blood culture specimens from inpatients at a University Hospital in Brazil, over a 20-year period by pulsed-field gel electrophoresis (PFGE) and multilocus sequence typing (MLST) techniques. The *ica* operon expression was shown in 83.6% strains, *bhp* gene in 11.5%, and *aap* gene in 32.8%. Oxacillin resistance was detected in 90.1%, while 4.9% showed tigecycline resistance, and intermediate resistance to quinupristin/dalfopristin was identified in 0.4%. Clonal profile determination showed 11 clusters, with the ST2 type determined as the major cluster. The *S. epidermidis* biofilm producer demonstrated a predominance of *agr* I locus, oxacillin resistance, and SCC*mec* III as well as the potential dissemination of pathogenic clones in hospital settings over long periods.

## 1. Introduction

Coagulase-negative Staphylococci (CoNS) are members of the genus *Staphylococcus* and major colonizers of the human microbiota. *Staphylococcus epidermidis* is the most commonly isolated microorganism in clinical materials and being opportunistic, it can cause serious infections, mainly in immunosuppressed patients and those who use prosthetics [1]. The ability to produce virulence factors, such as biofilm, and increased resistance to antimicrobials may make it difficult to treat infections caused by these microorganisms [2].

Biofilm, considered the main virulence factor of CoNS, is defined as a complex interaction of microorganisms incorporated into an extracellular matrix of polysaccharide, proteins, and nucleic acid [3], which confers protection to the involved microorganisms and prevent them from the host’s immune system action during infection [4]. Although the exact mechanism of biofilm formation has not yet been clarified, it is known that this process involves four steps: adhesion, accumulation, maturation, and detachment. In the constitution of the biofilm of *Staphylococcus* spp., some genes are involved in the coding of the most important substances and proteins: the *ica* operon, which carries the *icaA*, *icaC*, *icaD*, and *icaB* genes, the accumulation-associated protein (Aap), and the biofilm-associated protein homologue (Bhp) [3].

The operon *ica* synthesizes a polysaccharide intercellular adhesion (PIA) which allows cell-cell bonding and multilayer formation, its main function being the contribution to biofilm formation [3]. Despite its importance, it was recognized that the synthesis of PIA is not essential for the formation of biofilm in strains of *S. epidermidis* [5], because the formation of biofilm independent of PIA is related to proteins that can replace this polysaccharide, such as accumulation-associated protein (Aap) and biofilm-associated protein (Bhp).

The Aap protein is important for the accumulation and growth of the polymers that form the biofilm [5]. Aap is a protein with an N-terminal of domain A and one of domain B, processed by proteases, and its active form is a fibrillar protein, released in “tufts”. Its accumulation is through the dimerization of Zn2 + -dependent domain B on neighboring cells, while domain A is responsible for adhering to corneocytes, aiding adherence to the skin [3]. The Bhp protein is important as it promotes primary fixation on abiotic surfaces, as well as intercellular adhesion during biofilm formation. The Bhp protein is homologous to the Bap of *S. aureus*, and its mechanism of contribution to Biofilm formation is still unclear [5].

Biofilm formation, as well as other virulence factors in *Staphylococcus* spp., can be regulated by a system with quorum sensing activity, which allows communication between bacterial cells, the detection of cell density, and a phenotypic reaction according to the growth where the culture is located [6,7]. The accessory gene regulator (*agr*) is the main system with quorum sensing activity, and three polymorphisms of the *agr* locus (*agr* I, II, and III) are described in *S. epidermidis* [8]. The *agr* locus contributes to the regulation of the virulence of the CoNS at different stages of the infection, with adherence-related proteins produced in the exponential phase of the bacterial growth curve, and some exoproteins secreted in the post-exponential phase [9].

In addition to the biofilm formation capacity of CoNS, increasing antimicrobial resistance makes it difficult to treat infections caused by these bacteria. The high rates of oxacillin resistance highlight the importance of CoNS capable of forming a biofilm, since this is related to the persistence of infections and a decrease in the effectiveness of antimicrobial activity [3,10]. It has been suggested that the matrix of biofilms can be responsible for the increased resistance to antibiotics by acting as a diffusion barrier [11].

Oxacillin is a semi-synthetic penicillin used in Brazil as a sensitivity test and treatment for infections caused by *Staphylococcus* and 66% to 95% of clinical CoNS isolates are resistant to this drug [12]. Oxacillin resistance is mediated by the *mec*A gene, which encodes a supplemental penicillin-binding protein (PBP2a) that has low affinity for semi-synthetic penicillins. The *mec*A gene is located in a mobile genetic element identified as the staphylococcal chromosome cassette *mec* (SCC*mec*), composed of the *mec* complex, which encompasses the *mecA* gene and its regulators *mecI* and *mecRI*, by the complex of the *ccr* gene, responsible for the integration and excision of SCC*mec*, and the J region, which is not essential for the formation of SCC*mec*, but can carry genes that encode resistance to other non-β-lactam antimicrobials [13]. To date, 13 types of SCC*mec* (www.sccmec.org) have been identified, which are defined by the combination of the types of the *ccr* gene complex and the class of the *mec* gene complex, with the subtypes being defined by polymorphisms of the J region in the same combination of the *mec* and *ccr* complexes [13].

The objective of this study was to determine the *agr* and antimicrobial susceptibility of *S. epidermidis* isolated from blood culture capable of producing biofilm and to analyze the clonal profile of blood culture isolates from patients hospitalized over a period of 20 years.

## 2. Results

### 2.1. Identification of Strains

The CoNS identification by the biochemical method detected 223 (74.3%) *Staphylococcus epidermidis*, 27 (9.0%) *Staphylococcus haemolyticus*, 22 (7.3%) *Staphylococcus hominis*, 14 (4.7%) *Staphylococcus warneri*, nine (3.0%) *Staphylococcus lugdunensis*, and five (1.7%) *Staphylococcus capitis*. In contrast the genotypic technique of internal transcribed spacer by polymerase chain reaction (ITS-PCR) detected 223 (74.3%) *S. epidermidis*, 29 (9.7%) *S. haemolyticus*, 23 (7.7%) *S. hominis*, 11 (3.7%) *S. warneri*, nine (3.0%) *S. lugdunensis*, and five (1.7%) *S. capitis*. There was 98% agreement between the two methods used to identify the species of CoNS.

### 2.2. Detection of Biofilm Genes

We investigated the genes related to biofilm formation in the 300 isolates and the presence of *ica* operon genes and/or *bhp* and *aap* genes was detected in 163 (53.3%). The *icaA* gene was positive in 123 (41.3%) CoNS, the *icaB* in 165 (55.0%), the *icaC* in 222 (74.0%), and the *icaD* gene in 236 (78.7%). The presence of *icaA*, *icaB*, *icaC*, and *icaD* genes, concomitantly, was detected in 107 (35.7%) strains. We detected the *bhp* and *aap* genes in 37 (12.3%) and 104 (34.7%) of the CoNS, respectively. The distribution of these genes in relation to CoNS species can be visualized in Table 1.

### 2.3. Detection of mRNA of Biofilm Genes in CoNS Samples

The 163 strains that were positive for the operon *ica*, *bhp*, and *aap* genes were submitted to the reverse transcription polymerase chain reaction (RT-PCR) technique for detection of mRNA. Plankton cells in which the mRNA of the operon *ica* or the *bhp* or *aap* genes were detected were considered capable of producing biofilm. The capacity to produce biofilm was verified in 61 (37.4%) *S. epidermidis*. Of these 61 samples, the complete detection of *ica* operon was confirmed in 51 (83.6%) *S. epidermidis* and the mRNA of the *bhp* and *aap* genes was detected in seven (11.5%) and 20 (32.8%), respectively. There was concomitant expression of the *ica* operon and *bhp* gene in three *S. epidermidis* and the operon *ica* and *aap* gene in 12 *S. epidermidis*. There was no expression of genes related to biofilm formation in the other species studied.

### 2.4. Investigation of Biofilm Production by Adherence to Polystyrene Plates

**Figure 1:**
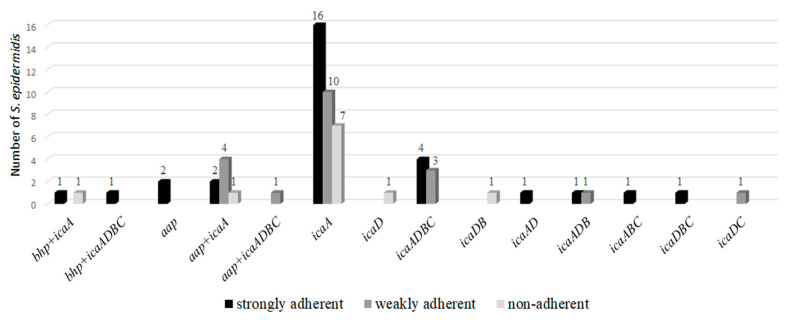
Correlation between adherence and expression of the detected genes.

The 61 *S. epidermidis* that presented mRNA for *icaA*, *icaD*, *bhp*, and/or *aap* genes were submitted to the adhesion technique on polystyrene plates to verify the production of biofilm. Strong adherence was observed in 49.0% *S. epidermidis*, weak adherence in 33% and non-adherence in 18.0% (Figure 1).

### 2.5. Determination of the agr Locus in Biofilm-Producing S. epidermidis

We investigated the *agr* locus (I, II, and III) in 61 *S. epidermidis* that expressed genes related to biofilm production. The *agr* I was detected in 47 (77.1%); 45.9% in those which expressed only the *ica* operon, 6.6% in those which expressed only the *aap* gene, 4.9% in those which expressed the *bhp* gene and *ica* operon, and 19.7% in those which expressed the *aap* gene and *ica* operon. The *agr* II was detected in 12 (19.6%) *S. epidermidis*, and also detected in all samples that expressed only the *bhp* gene. The *agr* locus was not found in two *S. epidermidis* that presented *ica* operon expression. These results are presented in Table 2.

### 2.6. Detection of mecA Gene and SCCmec in Biofilm-Producing S. epidermidis

We performed the *mecA* gene research on the 61 samples that confirmed the ability to produce biofilm by the RT-PCR technique and the *mecA* gene was detected in 55 (90.1%). The SCC*mec* typing determined five (9.0%) type I, 37 (60.6%) type III, and 14 (25.5%) type IV. The distribution of the *mecA* gene and the SCC*mec* types in the CoNS are shown in Table 3.

### 2.7. Determination of Minimum Inhibitory Concentration (MIC) of Antimicrobials in Biofilm-Producing S. epidermidis

We determined the MICs of the antimicrobials in the biofilm-producer *S. epidermidis* strains. Resistance to oxacillin was found in 83.6% of *S. epidermidis* and to tigecycline in 4.9%. However, intermediate resistance to quinupristin/dalfopristin was detected in one *S. epidermidis* presenting a MIC of 2 mg/mL for this drug. MIC values capable of inhibiting 50% and 90% of bacterial growth (MIC50 and MIC90) were determined, these values being considered indicators of oxacillin resistance. With the exception of oxacillin, vancomycin was the drug with the highest values of MIC50 and MIC90, but vancomycin resistance in *S. epidermidis* was not detected. The other drugs presented MIC values in the susceptibility range according to CLSI recommendations (2013). The results are shown in Table 4.

### 2.8. Determination of the Clonal Profile of the Biofilm-Producer S. epidermidis

Determination of the clonal profile of biofilm-producer *S. epidermidis* was performed by the pulsed-field gel electrophoresis (PFGE) technique. The macro-synthesis analysis of chromosomal DNA, using an established coefficient of 80% of similarity, allowed identification of the presence of 11 clusters, designated alphabetic letters from A to K. Four larger clusters were determined with 6–10 *S. epidermidis* isolates: A, G, H, and J.

Cluster A comprised six *S. epidermidis*, four of which were identical (100% similarity) and isolated over a period of 17 years, from 1993 to 2009, and all of them were positive for the *ica* operon, SCC*mec* III, and *agr*I. Cluster G grouped eight samples isolated from 2000 to 2009, all with SCC*mec* IV, *agr*I, and *ica* operon, and *bhp* in three isolates. Cluster H was the largest group, with 10 *S. epidermidis* isolated from 2001 to 2004, all of them positive for SCC*mec* III, *agr*I, and *ica* operon, and *aap* genes in three samples. Cluster J grouped six samples from the period 2000 to 2007, with *ica* operon, SCC*mec* III, and *agr*I.

**Figure 2:**
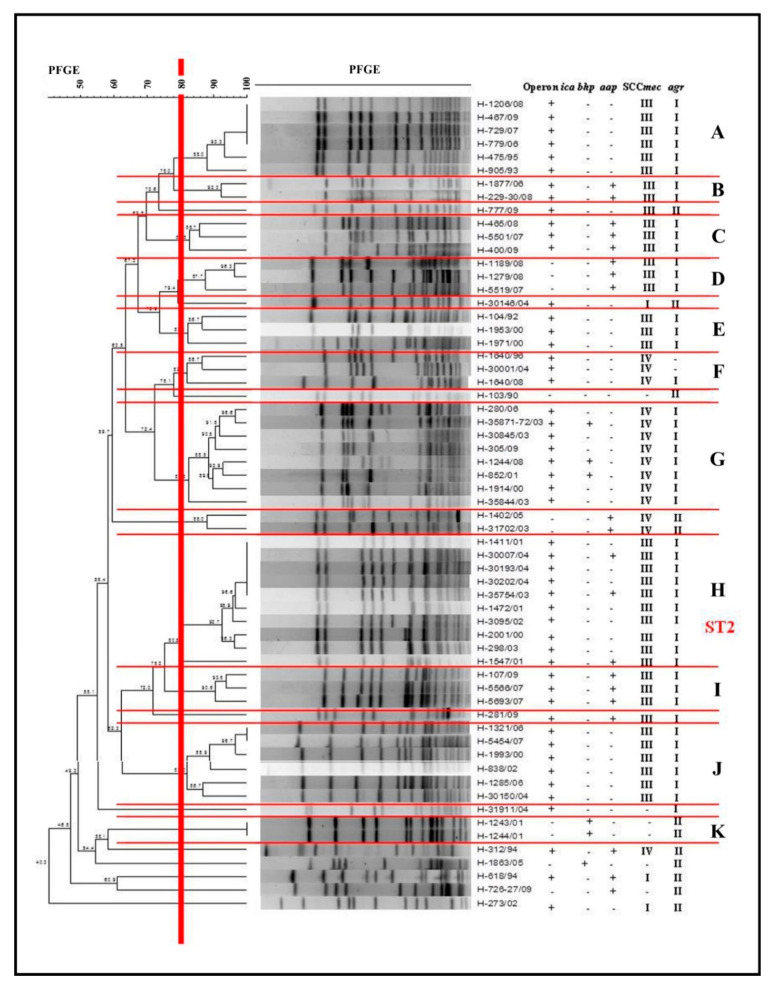
Dendrogram generated by the Dice/UPGMA (Bionumerics Applied Maths) analysis of *Staphylococcus epidermidis* biofilm producers isolated from 1990 to 2009, indicating the presence (+) or absence of (−) operon *ica* expression, *bhp* and *aap* genes, and carrying of the SCC*mec* and accessory gene regulator (*agr)* locus.

The other groups, including a smaller number of clones, were important, presenting isolated samples over long periods. Cluster E presented a single sample in 1992 and again in 2000, while cluster F presented isolated samples over a range of 12 years, with isolation in 1996, 2004, and 2008 (Figure 2).

We used the multilocus sequence typing (MLST) technique of Cluster H to characterize the largest number of isolates. A sample was selected and then submitted to the amplification and sequencing of the seven housekeeping genes, in order to obtain the type of sequence (ST). It was then characterized as ST2.

## 3. Discussion

Coagulase-negative staphylococci are opportunistic microorganisms capable of causing serious infections, mainly due to their ability of synthesizing biofilm, which protects the CoNS and increases the time spent in the hospital environment. In the current work, the capacity to produce biofilm was analyzed in 300 CoNS isolated from blood cultures, in addition to the possible mechanisms involved and susceptibility to antimicrobials used in the treatment of infections caused by these microorganisms.

The ability to adhere to surfaces and, consequently, to produce biofilm is the main virulence factors of CoNS, especially in *S. epidermidis*, which is the species most frequently isolated from clinical material [14]. The genes of *ica* operon were detected in all species studied, but the complete operon was detected in only 35.7% of the isolates, 98% of which belonged to the species *S. epidermidis*. The ability to produce polysaccharide intercellular adhesin (PIA), through the presence of mRNA, was detected only in the *S. epidermidis* strains, and the *ica* operon was expressed in 83.6%. Similar results were found in 2007 by Qin et al. [15], who detected *ica* genes in 21.8% of *S. epidermidis* from a group of 24 biofilm producing strains. These authors pointed out that the frequency of biofilm production and the presence of the *ica* operon are related to the different sources of lineages studied, such as sputum, blood cultures, catheters, and wounds. According to some studies, clinical isolates of *S. epidermidis* are those that most commonly present *ica* operon genes, as these genes are less frequent in CoNS isolated from healthy individuals, since the synthesis of PIA may require a great deal of adaptive cost, which is not advantageous for these bacteria [3].

It has recently been recognized that PIA is not essential for the formation of biofilm in *S. epidermidis*, since strains that did not have *ica* genes were isolated from biofilm-related infections. In some strains, biofilm formation may be additionally or exclusively mediated by proteins such as Bhp or Aap [16]. The presence of *bhp* and *aap* genes was also detected in our study, in 12.3% and 34.7% of the isolates, respectively. Although these percentages are lower than those presented by the *ica* operon, the genes were positive by the RT-PCR technique and were isolated or in concomitance with the *ica* operon, and the expression capacity was detected in 11.5% for the *bhp* gene and 32.8% for the *aap* gene. Similar results were found by Bowden et al. [17], who investigated these genes in clinical and nonclinical samples, determining the *bhp* gene in 9% of bacteremia isolates and 13% of skin colonizers, and the *aap* gene in 27% of bacteremias, 50% of contaminants in blood cultures, and 47% of *S. epidermidis* colonizers of the skin. The presence of the *bhp* and *aap* genes, concomitantly with the *ica* operon genes, suggests an association of the Bhp and Aap proteins with PIA, playing a role in intercellular adhesion and in the accumulation phase [17].

*S. epidermidis* that presented mRNA for *icaA*, *icaD*, *bhp* and/or *aap* genes were submitted to the adhesion technique on polystyrene plates to verify the production of biofilm. Strong adherence was observed in 49.0% *S. epidermidis*, weak adherence in 33% and non-adherent in 18.0%. The samples that expressed *icaA* (90.1%) were the ones that presented the strongest and weakest adherence. The expression of the *bhp* and *aap* genes were also important for and strong and weak adherence. The expression of the *icaD* gene not associated with *icaA* was less frequent (6.5%) and two samples were non-adherent. The environmental conditions or mechanism of gene regulation may have influenced the production of the protein and studies highlight the importance of the *icaA* gene in the production of staphylococcal biofilm and also of other genes, such as *bhp* and *aap*, in independent PIA strains [18,19].

The *agr* locus was investigated in *S. epidermidis* capable of producing biofilm, this being the most commonly detected *agr*I associated with the *ica* operon and *aap* gene. Strains that were positive for *bhp* gene expression showed *agr*II. As the regulation of the *agr* system is associated with cell density, these loci may be related to the release phases of biofilm cells, with the *agr*I and *agr*II loci being involved in this process [6,20]. *S. epidermidis* produces a series of exoproteases strictly regulated by the *agr* system, such as δ-toxin and other phenol-soluble modulins. These exoproteases act as surfactants, inhibiting the non-covalent bonds of the bacteria with the surface of the biofilm, resulting in detachment of the cells [3]. Studies have shown that in an *S. epidermidis agr* deletion mutant, increased biofilm development and intensified colonization in rabbits was due to non-regulation of exoproteins [21].

The *mec*A gene was positive in 90.1% of the biofilm-producer *S. epidermidis*, being present mainly in the samples that expressed the *ica* operon and *aap* gene and absent in those that expressed only the *bhp* gene. In the studies performed by Cabrera-Contreras et al. [22], the *mec*A gene was detected in 95% of *S. epidermidis* biofilm producers, and according to these authors, the presence of the *mec*A gene is enhanced in these samples, conferring resistance to the other β-lactam antibiotics. The SCC*mec* typing made possible to characterize SCC*mec* I, III, and IV in these samples, with SCC*mec* III being the most frequent. SCC*mec* III is the most commonly found element in multiresistant staphylococci related to nosocomial infections, since this element can carry other genes that confer resistance to other antimicrobial agents [23].

In addition to the *mec*A gene, the minimal inhibitory concentration of some antimicrobials used in the treatment of infections caused by CoNS was determined. Oxacillin presented 83.6% resistance, with MIC50 values above 256 mg/mL. The results also revealed 4.3% of tigecycline resistant samples and a sample with intermediate resistance to quinupristin/dalfopristin. Low MIC values were detected for linezolid and daptomycin, but higher MIC50 and MIC90 values for vancomycin were found. According to some authors, non-biofilm-producing bacteria are more susceptible to the action of antimicrobials and the host immune system. Therefore, the selective pressure imposed by the use of antimicrobials in the hospital environment favors the most adapted strains capable of producing biofilm [15,21]. Although there is an adaptive cost in the transport of genes related to biofilm formation and resistance to antimicrobials, a compensatory effect may occur through additional mutations favoring these microorganisms [24].

It is noteworthy that antimicrobial concentration values below the minimum inhibitory concentration (sub-MIC) can trigger an increase in the production of staphylococcus virulence factors, such as biofilm production. A study by Lázaro-Díez et al. [11] investigated the effects of subinhibitory concentrations of ceftaroline on the formation of biofilm by methicillin-resistant strains of *S. aureus* (MRSA) and found that sub-MIC of ceftaroline increased biofilm production by some strains of MRSA. Szczuka et al. [25] evaluated the sub-MICs of tigecycline and ciprofloxacin in strains of *S. epidermidis* and found that ciprofloxacin decreased biofilm formation, while tigecycline stimulated this process. The expression of *icaA* was increased by one-fold to 52-fold when the isolates were grown in the presence of 0.5 MIC tigecycline and 0.25 MIC resulted in an increase in *icaA* mRNA levels (by 2.6–12.6-fold) in three of the five isolates tested. These studies emphasize the importance of maintaining effective bactericidal concentrations of antimicrobials to combat infections related to *Staphylococcus* biofilm [11,25].

The clonal profile of the biofilm-producing *S. epidermidis* samples was established, with 11 clusters, including 4 larger clusters with a greater number of samples (6–10). The expression of the *ica* operon, *agr*I locus, and SCC*mec* III was found in clusters A, H, and J, and that of SCC*mec* IV in G. The potential to form biofilm favors these strains, which can colonize catheters, prostheses, and other materials for clinical use. This can cause invasive infections, and the proven multidrug resistance of these samples, as well as their ability to escape from the host’s immune system and the action of other antimicrobials, making it difficult to treat these infections. In addition, in the clusters mentioned above, as well as in the smaller clusters (E, F) the presence of these bacteria for long periods was verified, as it was possible to observe the same clone (A) over an interval of 17 years. The MLST analysis characterized the major cluster (H) as ST2. According to Otto [16], strains of *S. epidermidis* present a high level of diversity, and up to 74 types of sequences (STs) have been identified, the majority belonging to the clonal complex 2 (CC2), which includes the most frequently isolated ST2. The success of ST2 propagation may be due to the fact that all ST2s contain the IS256 insertion sequence and *ica* genes, factors that are correlated to invasive *S. epidermidis* isolates. In our study, ST2 isolates demonstrated biofilm formation capacity in vitro.

It is well established that biofilm-producing *S. epidermidis* are more pathogenic than non-biofilm producers, considered as commensal microorganisms. With the aim of better characterizing pathogenic strains of *S. epidermidis* isolated from clinical materials, studies suggest that the detection of biofilm expression capacity could be an excellent marker for the determination of invasive strains of *S. epidermidis* [18]. Biofilms can form on abiotic surfaces, such as surgical implants and catheters, and result in persistent infections that are difficult to treat, aggravating infections caused by *S. epidermidis* and increasing the length of stay of hospitalized patients.

## 4. Materials and Methods

### 4.1. Strains

A total of 300 strains isolated from blood cultures of patients hospitalized in the University Hospital of the Botucatu School of Medicine (HC-FMB) between 1990 and 2009 were studied. The strains were stored in the bacterial cultures collection of the Department of Microbiology and Immunology of the Institute of Biosciences, UNESP.

The strains were isolated as described by Koneman et al. [26] and coagulase- negative staphylococci were submitted to biochemical tests proposed in 2004 by Cunha et al. [27] for phenotypic identification of species.

Genotypic identification was performed using conserved sequence primers adjacent to the 16S and 23S genes for amplification of the ITS-PCR with the primers G1 and L1, as described by Barry et al. [28] and Couto et al. [29].

### 4.2. DNA Extraction

Total nucleic acid was extracted from *S. aureus* isolates cultured on blood agar, inoculated individually into brain-heart infusion broth, and incubated at 37 °C for 24 h. DNA extraction was performed using the Illustra kit (IllustraTM blood genomic Prep Mini Spin Kit), following the protocol described by Pereira et al. [30].

### 4.3. Detection of the Biofilm and mecA Gene

PCR reactions for the detection of genes related to biofilm formation (*icaA*, *icaB*, *icaC*, *icaD*, *aap*, and *bhp*) and the *mec*A gene were performed in microcentrifuge tubes of 0.5 mL in total volumes of 25 µL containing 10pmol of each primer (Table 5), 2.5 U of Taq DNA polymerase, 200 µM of deoxyribonucleotide triphosphate, 20 mM of Tris-HCl (pH 8.4), 0.75 mM of MgCl2, and 3 µL of DNA. The incubation was performed in a PTC-100 MJ research thermocycler, employing different parameters for each gene [31,32]. The efficiency of the amplifications was monitored by electrophoresis of the reaction in 2% agarose gel stained with Saber^TM^ Safe.

### 4.4. RNA Extraction and RT-PCR

Positive strains for genes related to biofilm formation were subjected to RNA extraction with the Illustra RNAspin Mini kit (GE Healthcare), following the manufacturer’s standards and the protocol described by Pereira et al. [30]. Total RNA was extracted from stationary phase planktonic cultures using the Illustra RNA spin Mini kit according to the manufacturer’s recommendations. After treatment with DNAse, the mRNA samples were converted into cDNA. For this purpose, 12 μL mRNA treated with DNAse was added to 1μL of random primer (75 ng/µL), 6 μL nuclease-free water, and 1 μL dNTP (200 μM). The mixture was heated for 5 min at 65 °C for RNA denaturation and primer binding and 4 µL reverse transcription buffer, 1 µL dithiothreitol and 1 µL SuperScript^TM^ III (200 U/μL) were added. cDNA was synthesized in a PTC-100 thermocycler using one cycle at 25 °C for 5 min, 50 °C for 60 min, and 70 °C for 15 min, followed by cooling at 4 °C. As internal control, the expression of 16S rRNA using 16S1 and 16S2 primers (Table 5) was analyzed, which corresponds to gene regions that are conserved in staphylococci and specific to the genus. The cDNA obtained was amplified by PCR and the resulting products were visualized by electrophoresis.

### 4.5. Investigation of Biofilm Production by Adherence to Polystyrene Plates

The quantitative method of adherence to polystyrene plates (TCP) proposed by Christensen et al. [36] was used in the present study, with modifications proposed by Oliveira and Cunha [14]. Trypticase soy broth (TSB) cultures were used, incubated for 24 h and then diluted 1:1 with TSB, prepared with 2% glucose. Sterile plates composed of 96 wells with a flat bottom (Costar, model 3599 manufactured by Corning Incorporated) were used. The wells were filled in quadruplicate with 200 μL of the diluted culture, using a multichannel pipette. International reference strains used as positive (*S. epidermidis* ATCC 35983) and negative controls (*S. epidermidis* ATCC 12228) and sterile TSB were included in all tests. The plates were incubated for 24 h at 37 °C and then the contents of each well were carefully aspirated using a multichannel pipette and then washed four times with 200 μL of phosphate buffered saline (PBS), pH 7.2. The plate was dried at room temperature for one hour. Then, the wells were stained with 2% violet crystal for one minute, and then the volume was aspirated, and the excess dye removed by washing the plates with distilled water using a multichannel pipette and subsequently dried at room temperature for 60 min, and the optical density reading was carried out in an ELISA reader (Labsystems, model Multiskan EX) using a 540-nm filter.

The isolates were classified into three categories: non-adherent, optical density equal to or lower than 0.111; weakly adherent, optical density higher than 0.111 or equal to or lower than 0.222; and strongly adherent, optical density higher than 0.222. When the cut-off corresponded to non-adherent, the isolates were classified as negative, and as positive when the cut-off corresponded to weakly or strongly adherent.

### 4.6. Determination of SCCmec

The SCC*mec* type was determined in *mec*A positive strains using multiplex PCR. The primers (Table 5) and parameters described by Oliveira et al. [34] and Machado et al. [37] were used for amplification.

### 4.7. Determination of Minimum Inhibitory Concentration by the E-Test

The susceptibility of the CoNS strains to the following antimicrobials were tested in vitro: oxacillin, vancomycin, daptomycin, linezolid, quinupristin/dalfopristin, and tigecycline. The MIC of these drugs was determined through the Etest^®^ (BioMérieux, Marcy l’Étoile, France). The procedure uses an inert plastic strip, which incorporates a stabilized concentration gradient of the antimicrobial to be investigated. First an inoculum of the sample was seeded following the 0.5 McFarland scale on a Mueller-Hinton plate and then the plastic strips containing each of the antimicrobials were applied to the inoculum and incubated at 35 °C for 24 h.

### 4.8. Determination of agr Group

All positive isolates from the RT-PCR technique for the detection of mRNA of genes related to biofilm were subjected to typing of the *agr* locus. The primers and parameters described by Li et al. [8] were used for amplification. The efficiency of the amplifications was monitored by electrophoresis of the reaction in 2% agarose gel stained with Saber^TM^ Safe.

### 4.9. Pulsed-Field Gel Electrophoresis

The positive isolates from the RT-PCR technique were submitted to clonal profile analysis through the PFGE technique. The clonal profile of the *Staphylococcus* spp. isolates was determined using the modified protocol of McDougal et al. [38], described by Pereira et al. [30].

BioNumerics software, version 6.1 (Applied Maths, Belgium), was used for analysis of similarity, calculation of the Dice correlation coefficient, and construction of the dendrogram by the UPGMA method (unweighted pair group method using arithmetic averages). Band position tolerance and optimization were set at 1.25% and 0.5%, respectively. A similarity coefficient of 80% was chosen for the definition of clusters.

### 4.10. Determination of Multilocus Sequence Typing (MLST)

MLST was performed according to standards described in 2000 by Enright et al. [35]. Each primer pair amplifies an internal fragment of the housekeeping gene (about 500 bp) (Table 5): carbamate kinase dehydrogenase (*arcC*), chiquimate dehydrogenase (*aroE*), glycerol kinase (*glpF*), guanylatoquinase (*gmk_*), phosphate acetyltransferase (*pta_*), triosephosphate isomerase (*tpi_*), and acetyl coenzyme A acetyltransferase (*yqiL*).

Purification was performed by the HiYield ™ Gel/PCR Fragments Extraction Kit and reactions were performed on an ABI3500 8 capillary (50 cm) sequencer using POP7 (Applied Biosystems) as the polymer. Sequence visualization (electrophoresis) was performed by Mega, Laser Gene and Bionumerics (version 7.1, Applied Maths, Belgium). The analysis and comparison of the sequences were performed on an internet database (http://www.mlst.net).

## 5. Conclusions

For the increased knowledge of the clones that persist in the Hospital das Clínicas of Botucatu, SP, Brazil over a period of 20 years, a characterization of multiresistance, the role ofthe *agr*I locus, and ability of biofilm formation in *S. epidermidis* strains isolated from blood culture is of great importance. Characterization of the major clone as ST2 highlights the relevance of these invasive samples in the hospital environment, emphasizing their potential to cause serious infections in hospitalized patients, and a high dissemination potential of these clones within the hospital environment.

Summary points:

- In some strains, biofilm formation may be additionally or exclusively mediated by proteins, such as Bhp or Aap.
- The *agr*I was associated with the *ica* operon and *aap* gene and strains that were positive for *bhp* gene expression showed *agr*II.
- The *mec*A gene was positive in 90.1% of the *S. epidermidis* capable of producing biofilm.
- The SCC*mec* typing made possible the characterization of SCC*mec* I, III, and IV in these samples, with SCC*mec* III being the most frequent.
- The expression of the *ica* operon, *agr*I locus, and SCC*mec* III was found in clusters A, H, and J, and that of SCC*mec* IV in G.
- The MLST analysis characterized the major cluster (H) as ST2.

## Figures and Tables

**Table 1:** Distribution of genes involved in the formation of Biofilm in Coagulase-negative Staphylococci (CoNS) species.

Species	*icaA*		*icaB*		*icaC*		*icaD*		Operon *icaADBC*		*bhp*		*aap*	
	N	%	N	%	N	%	N	%	N	%	N	%	N	%
*S. epidermidis*	119	96.8	144	87.3	181	81.5	179	75.8	105	98.1	34	91.9	103	99.0
*S. haemolyticus*	0	0.0	10	6.1	13	5.9	23	9.4	0	0.0	0	0.0	0	0.0
*S. hominis*	3	2.4	7	4.2	17	7.7	17	7.2	1	0.9	1	2.7	1	1.0
*S. warneri*	1	0.8	3	1.8	7	3.2	6	2.5	1	0.9	0	0.0	0	0.0
*S. lugdunensis*	0	0.0	0	0.0	2	0.9	7	3.0	0	0.0	1	2.7	0	0.0
*S. capitis*	0	0.0	1	0.6	2	0.9	4	1.7	0	0.0	1	2.7	0	0.0

N: number of samples; %: percentage of samples.

**Table 2:** Relationship of genes related to biofilm production and detection of accessory gene regulator (*agr*) locus in *Staphylococcus epidermidis* positive by the reverse transcription polymerase chain reaction (RT-PCR) technique.

		Locus *agr*		
	N	I	II	Not Detected
Operon *icaADBC*	34	28 (45.9%)	4 (6.5%)	2 (3.3%)
*bhp*	4	0 (0.0%)	4 (6.5%)	0 (0.0%)
*aap*	6	4 (6.6%)	2 (3.3%)	0 (0.0%)
Operon *ica* + *bhp*	3	3 (4.9%)	0 (0.0%)	0 (0.0%)
Operon *ica* + *aap*	14	12 (19.7%)	2 (3.3%)	0 (0.0%)

N = number of samples.

**Table 3:** Distribution of the *agr* locus, *mecA* gene, and Staphylococcal chromosomal cassette *mec* (SCC*mec*) in *S. epidermidis* positive by the RT-PCR technique.

	*mecA*	SCC*mec*			
		I	III	IV	Untyped
Operon *icaADCB* (*n* = 34)	33 (54%)	3 (5.5%)	22 (40%)	8 (14.5%)	0 (0.0%)
*bhp* (*n* = 4)	0 (0.0%)	0 (0.0%)	0 (0.0%)	0 (0.0%)	0 (0.0%)
*aap* (*n* = 6)	6 (9.8%)	1 (1.8%)	3 (5.5%)	2 (3.6%)	1 (1.8%)
Operon *ica* + *bhp* (*n* = 3)	3 (4.9%)	0 (0.0%)	0 (0.0%)	3 (5.5%)	0 (0.0%)
Operon *ica* + *aap* (*n* = 14)	13 (21.3%)	1 (1.8%)	12 (21.8%)	1 (1.8%)	0 (0.0%)

*n* = number of samples.

**Table 4:** Minimum inhibitory concentration (MIC) of antimicrobials (mg/mL), range of MIC (mg/mL), and percentage of resistant CoNS.

Antimicrobial	Breakpoint (Resistant)	MIC50	MIC90	Variation	Resistance
Oxacillin	≥0.5	>256	>256	0.047 to >256	83.6%
Vancomycin	≥32	1.5	2	0.125 to 2	0
Linezolid	≥8	0.25	0.38	0.094 to 1	0
Daptomycin	>1	0.125	0.19	0.019 to 0.5	0
Quinupristin/dalfopristin	≥4	0.19	0.5	0.094 to 2	0
Tigecycline	>5	0.125	0.25	0.016 to 0.75	4.9%

MIC50: Minimum antimicrobial inhibitory concentration to inhibit 50% of samples; MIC90: Minimum antimicrobial inhibitory concentration to inhibit 90% of samples.

**Table 5:** Oligonucleotides used in PCR techniques for genotypic identification, *mec*A gene, biofilm and SCC*mec* type.

Primer	Nucleotide Sequences (5′ to 3′)	Pb	Products	References
*agrA1*	GCTGCAACCAAGAAACAACC	1022	*agrI*, *II*, *III*	[8]
*agrA2*	CGTGTATTCATAATATGCTTCGATT			
*agrB1*	TATGCAAGCCAAGCACTTGT	453	*agrIII*	
*agrB2*	GTGCGAAAGCCGATAACAAT			
*agrC1*	CCTTGGCTAGTACTACACCTTC	615	*agrII*	
*agrC2*	GTGCTTGGCTTGCATAAACA			
*L1*	GAAGTCGTAACAAGG	-	16S	[28,29]
*G1*	CAAGGCATCCACCGT		23S	
*RNAr16S 1*	CCTATAAGACTGGGATAACTTCGGG	791	RNAr 16S	[33]
*RNAr16S 2*	CTTTGAGTTTCAACCTTGCGGTCG			
*icaA1*	TCTCTTGCAGGAGCAATCAA	187	IcaA	[31]
*icaA2*	TCAGGCACTAACATCCAGCA			
*icaB1*	CTGATCAAGAATTTAAATCACAAA	302	IcaB	
*icaB2*	AAAGTCCCATAAGCCTGTTT			
*icaC1*	TAACTTTAGGCGCATATGTTT	400	IcaC	
*icaC2*	TTCCAGTTAGGCTGGTATTG			
*icaD1*	ATGGTCAAGCCCAGACAGAG	198	IcaD	
*icaD2*	CGTGTTTTCAACATTTAATGCAA			
*bhp1*	ATGAAAAATAAACAAGGATTTC	1300	Bhp	
*bhp2*	GCCTAAGCTAGATAATGTTTG			
*aap1*	ATGGGCAAACGTAGACAAG	1100	Aap	
*aap2*	ACCGTAAAAATCGTAATTATCTC			
*mecA1*	AAAATCGATGGTAAAGGTTGG	533	PBP2a	[32]
*mecA2*	AGTTCTGCAGTACCGGATTTG			
DCS F2	CATCCTATGATAGCTTGGTC	342	SCC*mec* I, II e IV	[34]
DCS R1	CTAAATCATAGCCATGACCG			
CIF2 F2	TTCGAGTTGCTGGATGAAGAAGG	495	SCC*mec* I	
CIF2 R2	ATTTACCACAAGGACTACCAGC			
KDP F1	AATCATCTGCCATTGGTGATGC	284	SCC*mec* II	
KDP R1	CGAATGAAGTGAAAGAAAGTGG			
RIF4 F3	GTGATTGTTCGAGATATGTGG	243	SCC*mec* III	
RIF4 R9	CGCTTTATCTGTATCTATCGC			
arcC-Up	TTGATTCACCAGCGCGTATTGTC	456	Carbamate kinase	[35]
arcC-Dn	AGGTATCTGCTTCAATCAGCG			
aroE-Up	ATCGGAAATCCTATTTCACATTC	456	Chiquimate dehydrogenase	
aroE-Dn	GGTGTTGTATTAATAACGATATC			
aroE-Up	CTAGGAACTGCAATCTTAATCC	465	Glycerol kinase	
aroE-Dn	TGGTAAAATCGCATGTCCAATTC			
gmk-Up	ATCGTTTTATCGGGACCATC	429	Guanylate kinase	
gmk-Dn	TCATTAACTACAACGTAATCGTA			
pta-Up	GTTAAAATCGTATTACCTGAAGG	474	Phosphate acetyltransferase	
pta-Dn	GACCCTTTTGTTGAAAAGCTTAA			
tpi-Up	TCGTTCATTCTGAACGTCGTGAA	402	Triose-phosphate isomerase	
tpi-Dn	TTTGCACCTTCTAACAATTGTAC			
yqiL-Up	CAGCATACAGGACACCTATTGGC	516	Acetylcoenzyme A acetyl transferase	
yqiL-Dn	CGTTGAGGAATCGATACTGGAAC			
